# Effect of Glow Discharge Cold Plasma Treatment on the Physicochemical Properties and Antioxidant Capacity of Maize

**DOI:** 10.3390/foods14081312

**Published:** 2025-04-10

**Authors:** Miao Li, Chengcheng Ren, Caihong Li, Zengxuan Fan, Jiayin Zhu, Chenling Qu

**Affiliations:** Grain Storage and Security Engineering Research Center of Education Ministry, School of Food and Strategic Reserves, Henan University of Technology, Zhengzhou 450001, China; doublec0902@163.com (C.R.); licaihong0316@163.com (C.L.); f206998187@163.com (Z.F.); zhujiayin05777@163.com (J.Z.)

**Keywords:** color property, fatty acid, malondialdehyde, antioxidant enzymes, bioactive compounds, storage, enhancing effect

## Abstract

This study evaluated the effect of cold plasma (CP) on the physicochemical properties and antioxidant capacity of maize. CP treatments were performed using a glow discharge, applying argon and/or nitrogen at 50 W, with different working pressures (75, 100, and 125 Pa) and exposure times (1, 5, and 10 min). The maize samples were analyzed before and after treatments for color, fatty acid value (FAV), malondialdehyde content, superoxide dismutase and catalase activities, total phenol content (TPC), ascorbic acid content, reduced glutathione content, and antioxidant activity. The antioxidant activity was further evaluated during storage (25 °C for 180 days). After treatments, color parameters (brightness, yellowness, and saturation) showed measurable enhancement, while FAV and malondialdehyde content were significantly reduced by 14.95–56.37% and 11.38–43.71%, respectively. The optimal treatment conditions (100 Pa working pressure and 5 min exposure) maximized antioxidant enzyme activities and bioactive compound levels, accompanied by substantial increases in TPC. Under these conditions, maize samples had the highest organic radical scavenging capacities (DPPH), reaching 1.31-fold (argon plasma) and 1.25-fold (nitrogen plasma) that of untreated sample. During storage, all samples subjected to the optimal combined treatment exhibited higher DPPH radical scavenging capacity and ferric reducing antioxidant potential, along with lower FAVs and malondialdehyde contents compared to the untreated sample. Additionally, the DPPH radical scavenging capacity exhibited statistically inverse correlations with both FAV (r^2^ = −0.49) and malondialdehyde content (r^2^ = −0.15), as quantified through Pearson correlation analysis. These findings indicated that glow discharge cold plasma is a potentially effective non-thermal processing technique to enhance bioactive compound accumulation and antioxidant enzyme activity for preserving maize’s physicochemical properties, with possible use in the food industry for sustainable grain preservation strategies, particularly in delaying oxidative deterioration.

## 1. Introduction

As a predominant cereal crop globally, 12.42 billion tons of maize was produced in 2023, significantly exceeding rice (8.00 billion tons) and wheat (7.99 billion tons) production [1,2]. While its nutritional richness—70% starch, 10% protein, 5% lipid, and micronutrients [3]—positions maize as a critical resource for human and livestock sustenance [4], its high susceptibility to postharvest losses remains a pressing challenge. The grain’s hygroscopic embryo, high respiratory activity [5], and lipid peroxidation-prone polyunsaturated fatty acids [6] predispose it to fungal colonization and oxidative deterioration, exhibiting reduced storage stability compared with major cereal crops such as wheat and rice. This deterioration directly undermines food safety and economic viability, necessitating innovative interventions to preserve physicochemical integrity and enhance antioxidant defenses [7].

Cold plasma (CP), as an emerging non-thermal processing technology, has gained substantial research attention in agricultural, food, and biological applications due to its non-thermal nature, cost-effectiveness, operational versatility, and residue-free characteristics [2]. CP-generated reactive species (e.g., free radicals, molecules, atoms, charged particles, and photons) induce structural modifications in proteins accompanied by functional alterations [8,9], reshape starch architecture with consequent property changes [10,11], and suppress enzymatic activity associated with fruit/vegetable browning and ripening processes [12]. Recent applications in cereals demonstrate CP’s capacity to enhances cooking performance along with quality improvement in brown rice [13] and stimulate germination in wheat [14]. However, current research prioritizes fragmented optimization of processing parameters and seed viability traits while systematically neglecting holistic storage stability improvements encompassing lipid oxidation control and antioxidant preservation.

The majority of samples analyzed in previous cereal studies consist of protein isolates, pure starch, or flour. These materials exhibit greater susceptibility to degradation during routine storage than whole grains, particularly through lipid hydrolysis and oxidation processes. They fail to replicate the protective husk and lipid-rich germ of intact kernels—a disconnect that limits practical translation. Although CP treatment of intact kernels presents a more practical approach than the aforementioned sample types, existing research on grain plasma treatment has predominantly focused on two domains: surface decontamination and crop breeding [15,16]. Feizollahi et al. [17] demonstrated that atmospheric pressure cold plasma treatment reduces the deoxynivalenol contamination in barley while preserving its quality attributes. Subsequent studies have revealed that both low-pressure dielectric barrier discharge and low-frequency glow discharge plasma treatments significantly enhance the germination and growth performance of maize [18,19]. However, its systemic effects on whole-grain storage stability remain unexplored. The decline in the quality of cereals and their derived products is intricately linked to their antioxidant capacity, which is fundamentally determined by bioactive compound profiles including phenols, ascorbic acid, and GSH [20,21,22]. It is therefore imperative to elucidate the comprehensive effects of glow discharge cold plasma on the physicochemical properties and antioxidant capacity of maize.

The primary aim of this study is to systematically evaluate the effects of glow discharge cold plasma on maize storage stability, focusing on kernel coloration, fatty acid value, malondialdehyde content, antioxidant enzyme activities, and bioactive antioxidant retention. Distinct from prior plasma applications limited to seed decontamination or germination enhancement, this work pioneers a dual-functional preservation strategy targeting both structural integrity and oxidative defenses in intact maize kernels, thereby providing valuable insights for the application of this technology in maize preservation and addressing a critical gap in whole-grain storage technology.

## 2. Materials and Methods

### 2.1. Materials

The maize kernels (cv. Huanong 866; 11.5% moisture content) were harvested from experimental plots established using a randomized complete block design with three biological replicates [23]. The field trials were conducted in Xingyang City (34.88° N, 113.37° E), Henan Province, China, during October 2023. The following reagents were procured from Tianjin Komeo Chemical Reagent Co., Ltd., Tianjin, China: ethanol, sodium hydroxide, phenolphthalein, trichloroacetic acid, sodium carbonate, ascorbic acid, metaphosphoric acid, oxalic acid, ethylene diamine tetraacetic acid (EDTA), ammonium molybdate, potassium phosphate and glacial acetic acid. 2-thiobarbituric acid, Folin–Ciocalteu phenol reagent, and 1,1-diphenyl-2-picrylhydrazyl radicals were purchased from Shanghai Aladdin Biochemical Technology Co., Ltd., Shanghai, China, while gallic acid and 5,5′-dithiobis (2-nitrobenzoic acid) (DTNB) were provided by Shanghai Yuanye Biotechnology Co., Ltd., Shanghai, China. All reagents were of analytical grade and used without any further purification.

### 2.2. Plasma Treatment and Maize Storage

A total of 1000 g of maize kernels was weighed and placed in the cold plasma seed treatment device (HD-3N, Zhongke Changtai Plasma Technology Co., Ltd., Changzhou, China), as previously described [24]. To minimize high-energy particle bombardment on seeds during plasma discharge, treatment parameters were optimized based on previous studies and preliminary experiments [25]. Following the configuration of the power output to 50 W and the vacuum to 10 Pa, argon or nitrogen was introduced, and the treatment was performed at varying working pressures (75, 100, and 125 Pa) for differing durations (1, 5, and 10 min). Each treated sample was labeled as X-Y m, where X represents the working pressure and Y represents the treatment time. Untreated samples (0 min) under each gas source were designated as controls.

A 100 g quantity of each maize kernel sample, both before and after treatment, was used to analyze the color attributes. Additionally, 400 g of each maize kernel sample was ground into powder using a low-temperature continuous hammer cyclone mill (TDW 5000, Tongxin Tianbo Technology Development Co., Beijing, China) and subsequently stored at 4 °C for further analysis. The remaining kernels from all maize samples were stored in airtight polyethylene bags (0.16 mm thickness) for 180 days at 25 ± 1 °C. During the storage period, the seeds in each bag were mixed periodically. At 30-day intervals, an appropriate quantity of kernels was collected and designated as a test sample.

### 2.3. Color Characteristics

A colorimeter (CR-410, Hangzhou Ke Sheng Instrument Co., Ltd., Hangzhou, China) was used to measure the color parameters (L*, a*, and b*) of maize kernels after treatment. The total color difference (Δ*E*), chroma (C*), and hue (h*) were calculated according to the following equations [26]:

$$∆E=\sqrt{{\left(L_{0}*-L*\right)}^{2}+{{(a}_{0}*-a*)}^{2}+{{(b}_{0}*-b*)}^{2}}$$

$$C^{*}=\sqrt{a*+b*}$$

$$h*=\tan^{-1}\left(\frac{b*}{a*}\right)$$

The values of $L_{0}*$, $a_{0}*$, and $b_{0}*$ were determined from the untreated control samples, representing the lightness (100: perfect white; zero: black), greenness-redness, and blueness-yellowness, respectively [27]. All images were captured using a Canon EOS R6 camera (Canon Inc., Tokyo, Japan) positioned at a vertical distance of 10 cm from the corn kernel samples and stored in JPEG format.

### 2.4. Fatty Acid Value (FAV)

FAV was determined following Liu’s method [28], with minor modifications. Briefly, 1 g of maize kernel powder was mixed with 6 mL of 95% ethanol (*v*/*v*) and vortexed thoroughly for 1 h. The mixture was then centrifuged at 3000 g for 5 min at room temperature. Subsequently, 4 mL of the resulting supernatant was collected, and five drops of phenolphthalein solution were added as an indicator. The mixture was titrated with 0.05 M sodium hydroxide until a pink color persisted for a period of 10–15 s. Moreover, 4 mL of 95% ethanol was utilized as a blank experiment, in lieu of the 4 mL of the preceding sample. FAV was expressed as mg NaOH/100 g, representing the amount of NaOH required to neutralize free fatty acids in 100 g of maize kernel powder.

### 2.5. Malondialdehyde (MDA) Content

The MDA content in all maize powder samples was analyzed using spectrophotometry based on the thiobarbital method [29]. The samples were mixed with 10% (*w*/*v*) trichloroacetic acid, vortexed vigorously, and then subjected to centrifugation (8000 g, 15 min) at 4 °C. The resulting supernatant (upper layer) was collected as the MDA extract. The extract was then reacted with 0.67% (*w*/*v*) 2-thiobarbituric acid in a boiling bath and centrifuged again after rapid cooling. The supernatant was collected and absorbances at 450, 532, and 600 nm were measured. Results were expressed as µmol/g on a fresh weight basis.

### 2.6. Enzyme Activities of Superoxide Dismutase (SOD) and Catalase (CAT)

The activities of SOD and CAT were measured spectrophotometrically using colorimetric assay kits (Catalog #BC0170 for SOD and Catalog #BC0200 for CAT; Beijing Solarbio Science & Technology Co., Ltd., Beijing, China) according to the manufacturer’s protocols. For both enzymatic analyses, precisely 0.1 g of each maize powder sample was used to obtain crude enzyme extracts. Those extracts were subsequently analyzed using a UV-Vis spectrophotometer (UV-3400S, Shanghai Linchylab Instruments Co., Ltd., Beijing, China), with absorbance measurements recorded at specific wavelengths: 560 nm for SOD and 240 nm for CAT. All results were expressed in U/g on a fresh weight basis.

### 2.7. Total Phenol Content (TPC)

The TPC of maize powder samples, both before and after CP treatment, was determined using the Folin–Ciocalteu method, as described by Spanos and Wrolstad [30]. Measurements were conducted with a UV-Vis spectrophotometer (UV-3400S, Shanghai Linchylab Instruments Co., Ltd., Beijing, China) at a wavelength of 765 nm. The TPC was calculated according to the calibration curve (y = 0.0034x + 0.0032, R^2^ = 0.9996) derived from a 0.5 mg/mL gallic acid standard solution. The results were represented as gallic acid equivalents (mg/g) in the maize powder samples.

### 2.8. Ascorbic Acid (AA) Content

The modified molybdenum blue colorimetric method, as referenced in prior research [31], was used in the analysis. Briefly, 0.1 g of maize powder was thoroughly mixed with 5 mL of oxalic acid–EDTA solution in a mortar to obtain a homogeneous mixture. This mixture was then transferred to a centrifuge tube and centrifuged at 3000 rpm for 10 min. After centrifugation, 1 mL of the supernatant was carefully collected and transferred to a fresh centrifuge tube. Subsequently, 3 mL of oxalic acid–EDTA solution, 0.8 mL of 5% (*v*/*v*) sulfuric acid, 0.4 mL of metaphosphoric acid–acetic acid solution, and 1.6 mL of 5% (*w*/*v*) ammonium molybdate solution were added sequentially. The resulting mixture was vortexed vigorously to ensure complete homogenization and then incubated in a water bath at 30 °C for 10 min. The mixture’s absorbance was measured at a wavelength of 760 nm. A calibration curve (y = 0.2896x − 0.0042, R^2^ = 0.9992) was prepared using a serially diluted ascorbic acid standard solution (1 mg/mL) with concentrations ranging from 0.4 to 5.6 mg/L. The AA content was calculated based on the standard curve and expressed as mg/100 g on a fresh weight basis.

### 2.9. Glutathione (GSH) Content

The GSH content in maize kernel was determined using Ellman’s method [32]. Briefly, 0.3 g of maize powder was mixed with 5% (*w*/*v*) metaphosphoric acid solution in a mortar to form a homogeneous mixture, which was then adjusted to a total volume of 10 mL with the same acid solution. The mixture was centrifuged at 2000 rpm for 10 min at 4 °C. Subsequently, 2 mL of the supernatant was collected and transferred to a fresh centrifuge tube. Then, 4 mL of potassium phosphate-buffer solution and 0.4 mL of DTNB solution were added to the supernatant. The mixture was vortexed thoroughly and left to react at room temperature for 20 min. The resulting mixture was then subjected to a spectrophotometric analysis at a wavelength of 412 nm, and the GSH content was subsequently calculated using a calibration curve (y = 0.0063x − 0.002, R^2^ = 0.9974) derived from a 10 μg/mL GSH standard solution. The results were expressed as μg/g on a fresh weight basis.

### 2.10. Antioxidant Capacity

The antioxidant capacity was assessed using the 1,1-diphenyl-2-picrylhydrazyl (DPPH) free radical scavenging method [29] and the ferric reducing antioxidant power (FRAP) method. The FRAP assay was conducted using a total antioxidant capacity assay kit (Catalog #BC1310) purchased from Beijing Solarbio Science & Technology Co., Ltd. (Beijing, China). For the DPPH assay, 200 mg of each maize powder sample was used, whereas 100 mg of each sample was used for the FRAP assay. Each assay was performed in triplicate. The results were expressed as the scavenging percentage for the DPPH assay and as μmol/g on a fresh weight basis for the FRAP assay.

### 2.11. Statistical Analysis

All experiments were conducted in triplicate and the results were expressed as mean ± standard deviation. Statistical significance was determined by one-way ANOVA, followed by the Duncan multiple test model (*p* < 0.05) using SPSS 26.0 software (SPSS Inc., Chicago, IL, USA). Prior to ANOVA, data normality and homogeneity of variance assumptions were verified using SPSS. A correlation heatmap was plotted with Origin 2022 software (OriginLab Corporation, Northampton, MA, USA).

## 3. Results and Discussion

### 3.1. Color Evolution of Maize Kernels

As a critical quality indicator for agricultural products, color is always linked to freshness and has a direct impact on consumer preferences [33]. Lightness (L*) and yellowness (b*) values were employed as indicators to assess corn freshness [34], with higher values corresponding to increased brightness and yellowness. In addition, chroma values (C*) exhibited a positive correlation with perceived color intensity in human visual assessment [26].

**Figure 1:**
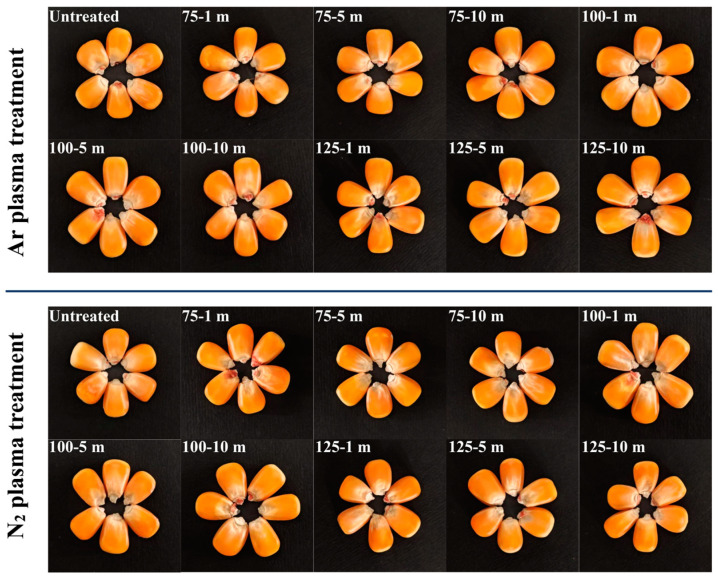
Effect of CP treatments on overall appearance of maize kernels.

As demonstrated in Figure 1 and Table 1, cold plasma (CP) treatment modifies the color characteristics of maize kernels. In comparison to untreated controls, all treated samples exhibited increased L* and b* values, with decreased a* values, suggesting that CP treatment enhances kernel brightness while intensifying yellowness and attenuating redness. A significant elevation in chroma values (C*) was observed between treated samples and untreated controls (*p* < 0.05), demonstrating enhanced color saturation induced by CP processing. Ar plasma-treated samples showed a slight increase in hue (h*) values, whereas N_2_ plasma-treated samples exhibited significantly higher h* values compared to the untreated control (*p* < 0.05). The elevated h* values indicated a shift in maize hue from redness to yellowness following CP treatment. Notably, the 100-5 m samples achieved the highest L*, b*, C*, and h* values, along with the lowest a* values.

Previous studies have demonstrated that carotenoids determine the color intensity of orange and yellow maize kernels [35], with the carotenoid concentration being inversely correlated with the b* value [36]. Further studies have indicated that the characteristic red intensity imparted by carotenoids in food products exhibits a positive correlation with the number of conjugated double bonds, as a higher count of conjugated double bonds leads to increased absorption maxima (λ_max_) [37,38]. In this study, the enhanced yellowness and reduced redness of maize kernels were mechanistically attributed to diminished carotenoid concentrations, resulting from (i) cold plasma-emitted UV light-mediated photo-oxidation of carotenoids and (ii) reactive nitrogen–oxygen species-driven β-carotene degradation/isomerization during CP processing.

Meanwhile, plasma-induced surface etching, inhibition of polyphenol oxidase and peroxidase activity, and carotenoid degradation through conjugated double-bond cleavage likely explain the L* value elevation [39,40,41,42]. Except for the 100-5 m sample exposed to N_2_ plasma, the other treated samples did not exhibit significant color variations from their untreated controls, as indicated by their Δ*E* values being below the threshold of 5 [43]. Consequently, CP treatment increased brightness, yellowness, and color saturation, indicating that CP could effectively preserve the freshness of maize kernels. These color alterations influenced consumer perception and sensory acceptability by maintaining visual quality traits closely associated with maize freshness. Previous studies that have documented CP-induced color brightening in green peas [44] and brown rice [40] were consistent with our results, demonstrating the effectiveness of CP in maintaining the marketability of maize.

### 3.2. Fatty Acid Value (FAV) Change and Malondialdehyde (MDA) Accumulation

FAV exhibits a strong correlation with free fatty acid content, which originates from triglyceride hydrolysis and undergoes oxidative rancidity, ultimately accelerating quality deterioration in agricultural products [45,46]. MDA, as the primary product of membrane lipid peroxidation, serves as a widely accepted biomarker for quantifying oxidative damage intensity in stored grain cells [47].

**Figure 2:**
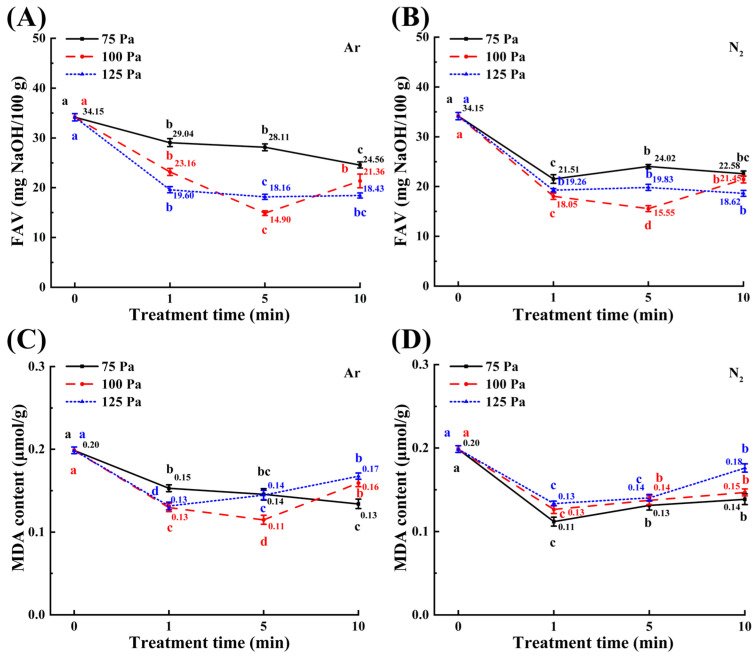
Effect of CP treatments on FAV (**A**,**B**) and MDA content (**C**,**D**) in maize kernels. Different colored letters indicate significant differences (*p* < 0.05) between samples at each working pressure.

As demonstrated in Figure 2A,B, all CP treatments significantly reduced FAV and MDA levels in maize kernels (*p* < 0.05). Among all treated samples, the 100-5 m samples exhibited the lowest FAVs, at 14.90 mg NaOH/100 g for Ar plasma treatment and 15.55 mg NaOH/100 g for N_2_ plasma treatment (*p* < 0.05) (Figure 2A,B). This reduction likely stems from oxidative interactions between CP-generated reactive species (e.g., electrons, ions) and unsaturated fatty acids (e.g., oleic acid, linoleic acid), thereby diminishing their contents [48]. Concurrently, microorganisms in stored grain secrete enzymes that cause hydrolysis reactions, thereby forming free fatty acids [49]. This suggests that CP’s antimicrobial efficacy may synergistically contribute to FAV reduction [50].

As shown in Figure 2C,D, the MDA contents of maize samples after all CP treatments were 57.58–84.42% (Ar plasma) and 56.28–88.44% (N_2_ plasma) of untreated controls. Minimal MDA accumulation was observed in samples 100-5 m (Ar plasma) and 75-1 m (N_2_ plasma). Furthermore, the MDA content in the treated maize samples demonstrated a general trend of decreasing initially and then increasing over time, with the exception of those treated with Ar at 75 Pa working pressure. This phenomenon can be attributed to the exposure of maize to prolonged and intense plasma treatment, which has been shown to induce a state of stress, resulting in a slight increase in MDA content [51]. The observed reduction in FAV and MDA content principally originates from plasma-generated reactive species interfering with lipid oxidation cascades. Lipoxygenase catalyzes the oxidation of polyunsaturated fatty acids liberated through lipase-mediated hydrolysis of membrane phospholipids, driving their conversion into lipid hydroperoxides [52]. The latter decomposes to produce secondary oxidation products such as MDA with shorter carbon chains [53]. Research indicates that reactive nitrogen–oxygen species generated by CP interact with those enzymes through hydrogen bonding, leading to structural changes and final inactivation [54,55,56]. The coordinated attenuation of FAV and MDA across treatments underscores CP’s capacity to mitigate cellular oxidative injury and inhibit lipid peroxidation cascades under suitable treatment conditions.

### 3.3. Superoxide Dismutase (SOD) and Catalase (CAT) Activity Analysis

**Figure 3:**
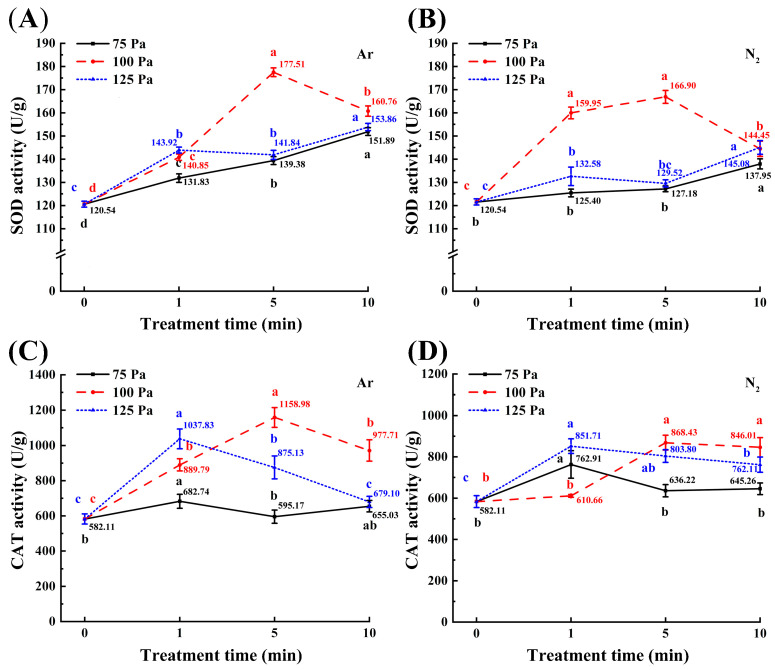
Effect of CP treatments on SOD (**A**,**B**) and CAT (**C**,**D**) activity in maize kernels. Different colored letters indicate significant differences (*p* < 0.05) between samples at each working pressure.

SOD and CAT, as the most important enzymatic antioxidants, protect plant cells from being oxidized by O_2_^−^ [57] and damaged by H_2_O_2_ [58], respectively. As given in Figure 3, the SOD and CAT activities of all treated maize samples were obviously higher than those of untreated controls. Under Ar plasma treatment, the 100-5 m sample demonstrated maximal enzymatic enhancement, with SOD and CAT activities reaching 1.47-fold and 1.99-fold of untreated control levels (*p* < 0.05), respectively. A similar outcome was observed for N_2_ plasma treatment, where the 100-5 m sample attained peak activities of 166.90 U/g (SOD) and 868.43 U/g (CAT) (*p* < 0.05). These findings align with documented plasma-induced antioxidant activation in blueberries [59], cumin seedlings [60], and *Astragalus membranaceus* seedlings [61], collectively demonstrating that CP treatment under appropriate conditions enhance oxidative stress resilience by stabilizing enzymatic antioxidant systems in plant tissues [62]. The increase in SOD and CAT activity in maize samples may be attributable to the upregulation of SOD- and CAT-related genes, which was induced by an increase in endogenous reactive oxygen species (ROS) resulting from the production of exogenous ROS after CP treatments [18,62].

### 3.4. Changes in Total Phenol, Ascorbic Acid (AA), and Glutathione (GSH) Content

Previous research has reported that non-enzymatic antioxidants, together with enzymatic antioxidants in cereals and fruits, play an important role in the scavenging of intracellular ROS induced by biotic and abiotic stresses, such as fungal infection [63], UV-B radiation [64], NaCl stress [65], and heat treatment [66]. To evaluate CP effects on maize antioxidant systems, we systematically analyzed the changes in total phenol, AA, and GSH contents before and after treatment.

**Figure 4:**
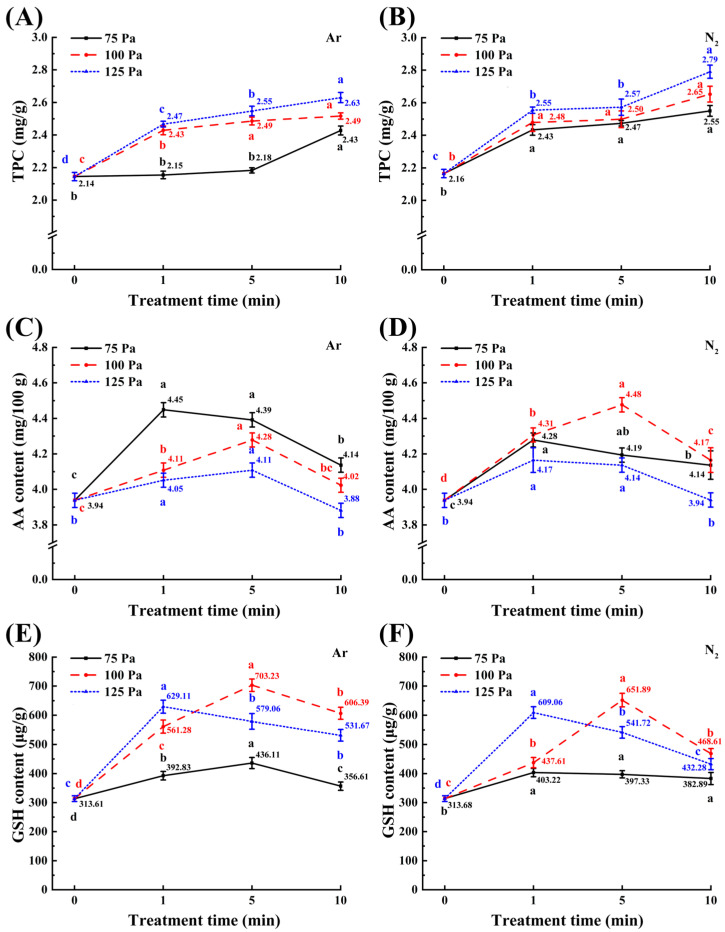
Effect of CP treatments on the contents of total phenols (**A**,**B**), AA (**C**,**D**) and GSH (**E**,**F**) in maize kernels. Different colored letters indicate significant differences (*p* < 0.05) between samples at each working pressure.

As can be seen from Figure 4A,B, total phenol content (TPC) exhibited a progressive increase with elevated working pressure and extended treatment time. The maximal enhancement of TPC was observed in the 125-10 m samples, reaching 2.63 mg/g (Ar plasma) and 2.79 mg/g (N_2_ plasma), which represent significant increases in comparison to the untreated controls (2.14 mg/g and 2.16 mg/g, respectively; *p* < 0.05). This result is probably related to reduced TPC utilization via lower activities of polyphenol oxidase and peroxidase, both of which use phenolic compounds as substrates to counteract oxidants [67]. Deactivation of both polyphenol oxidase and peroxidase was found to occur as a result of the plasma-generated reactive species interacting with the amino acids and secondary structure of these enzymes [55]. The observed sustained elevation in TPC under intensified plasma conditions (higher working pressure and prolonged treatment time) can be attributed to the depolymerization of polyphenols [68].

As shown in Figure 4C–F, the AA and GSH contents in maize samples exhibited an initial increase followed by a subsequent decrease with prolonged treatment time across all plasma treatments at constant working pressure. Notably, the Ar and N_2_ plasma-treated samples, specifically 75-1 m and 100-5 m, demonstrated a significant and maximal enhancement in AA content, showing increases of 12.95% and 13.67%, respectively, compared to their untreated controls (*p* < 0.05). Furthermore, the application of Ar and N_2_ plasma treatments resulted in a significant increase in GSH content in the maize samples (*p* < 0.05). Among these, the 100-5 m samples showed the highest GSH levels, which were 2.24-fold and 2.08-fold higher than those of their respective untreated controls. This observed increase may be due to changes in enzyme activity associated with GSH metabolism following CP treatment [69].

### 3.5. Antioxidant Capacity Analysis

**Figure 5:**
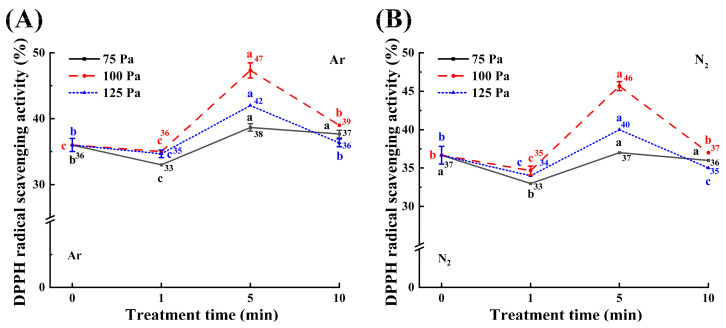
Effect of CP treatments ((**A**) Ar plasma; (**B**) N_2_ plasma) on DPPH radical scavenging activity in maize kernels. Different colored letters indicate significant differences (*p* < 0.05) between samples at each working pressure.

The results presented above demonstrate that CP treatment effectively enhances antioxidant enzymes and compounds in maize, thereby mitigating oxidative damage. In order to further evaluate the antioxidant capacity of the maize samples before and after treatment, the 1,1-diphenyl-2-picrylhydrazyl (DPPH) assay was employed (Figure 5). The trends in DPPH radical scavenging activity of maize kernels treated with Ar and N_2_ plasma were comparable. During the initial treatment phase (1 min), scavenging activity decreased across all working pressures, likely due to induced oxidative stress. However, when the treatment time was extended to 5 min, a significant increase in scavenging activity was observed in treated samples compared to their respective untreated controls. The most pronounced enhancement occurred at 100 Pa working pressure, exceeding the effects observed at 125 Pa and 75 Pa. Specifically, the 100-5 m samples treated with Ar and N_2_ plasma demonstrated the greatest improvement, reaching 1.31-fold and 1.25-fold increases, respectively, compared to untreated controls. These findings suggest that moderate working pressure optimizes the antioxidant capacity of maize, enabling samples to adapt to treatment conditions and counteract oxidative stress. Although scavenging activity declined in all treated samples after 10 min of treatment, samples treated at 100 Pa maintained the highest scavenging activity, confirming that appropriate working pressure and treatment time are crucial for enhancing antioxidant capacity.

**Figure 6:**
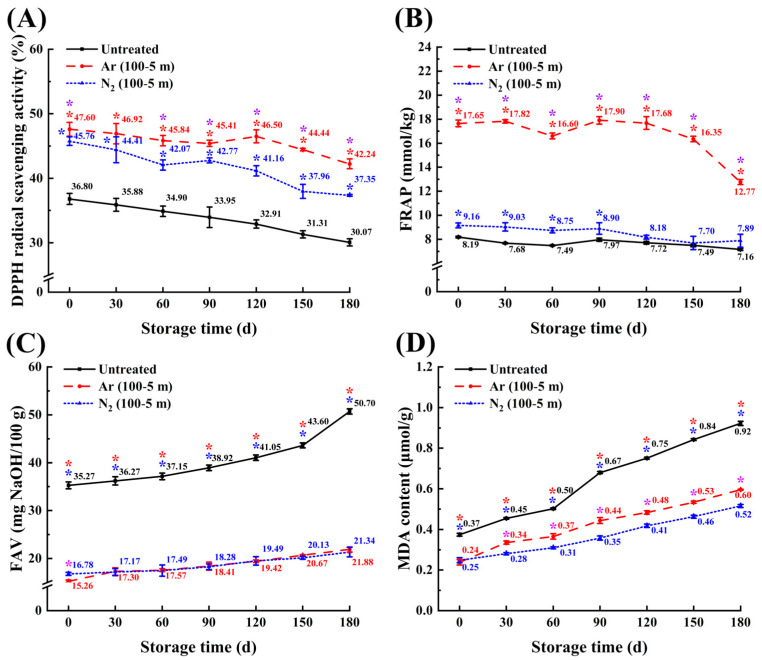
Effect of CP treatments on DPPH radical scavenging activity (**A**), FRAP (**B**), FAV (**C**), and MDA content (**D**) in maize kernels during storage for 180 d at 25 °C. */*/* represent comparisons between all treated samples with Ar plasma and the untreated control, comparisons between all treated samples with N_2_ plasma and the untreated control, and comparisons between samples treated with different gas plasma at each time, respectively (*p* < 0.05).

To further elucidate the role of CP treatment in enhancing maize antioxidant capacity during storage, the 100-5 m samples treated with Ar and N_2_ plasma, along with untreated controls, were stored for 180 days at 25 °C. The antioxidant properties were monitored using DPPH and ferric reducing antioxidant potential (FRAP) assays. As shown in Figure 6, all maize samples exhibited a gradual decline in antioxidant activity, with fluctuations observed in both DPPH and FRAP measurements. This phenomenon may be attributed to the prolonged depletion of non-enzymatic antioxidants during storage, which are continuously consumed to neutralize reactive oxygen species generated by metabolic activities in maize kernels, while also serving as substrates for oxidative catalysis by polyphenol oxidase and peroxidase, collectively leading to their degradation and diminished antioxidant capacity [67,70]. Furthermore, progressive lipid oxidation during storage results in the accumulation of MDA, which likely inactivates SOD through chelation of Cu^2+^ ions at its catalytic active sites, thereby disrupting enzymatic antioxidant defense mechanisms [71].

Compared to the untreated controls, CP-treated samples demonstrated significantly enhanced DPPH scavenging activity throughout storage (*p* < 0.05) (Figure 6A). Notably, Ar plasma-treated samples consistently maintained higher antioxidant activity than N_2_ plasma-treated samples, except at day 30. Furthermore, FRAP analysis revealed that the 100-5 m samples exhibited higher values than the untreated control (Figure 6B). Significant differences in FRAP values were observed between Ar plasma-treated samples and untreated control throughout storage, while N_2_ plasma-treated samples showed significant differences from the untreated control from day 0 to day 90 (*p* < 0.05). Furthermore, the FRAP values of the Ar plasma-treated samples (100-5 m) were consistently and significantly higher than those of N_2_ plasma-treated sample throughout the storage period. These results demonstrate that CP treatment with Ar plasma provides a stronger enhancement of antioxidant capacity compared to N_2_ plasma, further supporting the potential of CP treatment under appropriate conditions to improve the antioxidant properties of maize during storage.

### 3.6. The Recovery of FAV and MDA Content During Storage

Figure 6C,D present the changes in FAV and MDA content of the 100-5 m samples and the untreated control during a 180-day storage period. Both FAV and MDA content exhibited a gradual increase in all maize samples throughout storage. The untreated control exhibited a rapid escalation in these parameters, with FAV increasing from 35.27 to 50.70 mg NaOH/100 g and MDA content rising from 0.22 to 0.97 μmol/g over the storage period. In contrast, the 100-5 m samples maintained significantly lower FAV and MDA levels compared to the untreated control at each time (*p* < 0.05). At the end of the storage period (day 180), the Ar plasma-treated sample (100-5 m) demonstrated FAV and MDA contents that were 43.15% and 60.84% of the untreated control, respectively. Similarly, the N_2_ plasma-treated sample (100-5 m) showed FAV and MDA contents corresponding to 42.09% and 44.02% of the untreated control, respectively. These findings are consistent with previous studies demonstrating that CP treatment effectively reduces FAV in brown rice and MDA content in foxtail millet under appropriate treatment conditions during storage [72,73]. It is noteworthy that the Ar plasma-treated sample exhibited significantly higher MDA content than the N_2_ plasma-treated sample throughout the storage period, a phenomenon potentially attributable to the distinct characteristics of reactive species generated by different plasma gases [74]. These results, combined with the observed enhancement of antioxidant capacity, suggest that CP treatment under appropriate conditions can effectively delay maize aging by significantly mitigating lipid peroxidation.

### 3.7. Correlation Analysis

**Figure 7:**
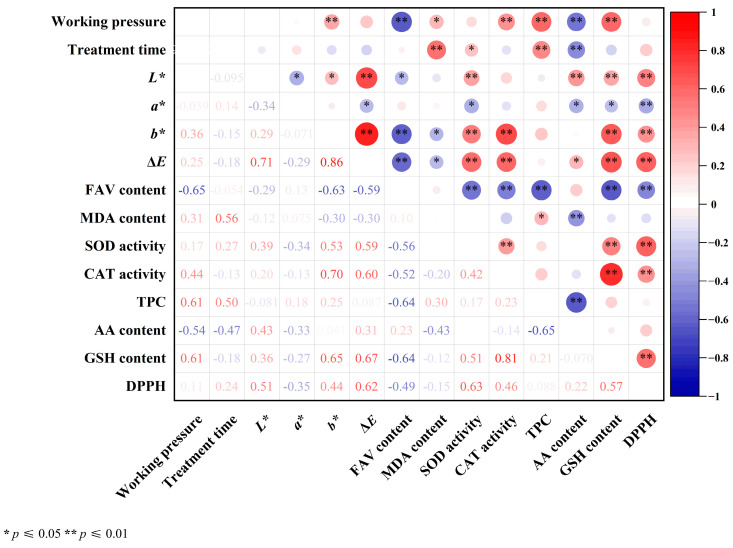
Correlations between changes in color, FAV, MDA, antioxidant enzyme activity, antioxidant compounds, and DPPH scavenging activity in maize kernels. * and ** indicated significant differences at the *p* ≤ 0.05 and *p* ≤ 0.01 levels, respectively. Red (+1) and blue (−1) colors show the positive and negative correlations between different indexes, respectively.

In order to comprehensively comprehend the effects of CP on the physicochemical properties and antioxidant capacities of maize kernels, a correlation analysis was conducted between color (L*, a*, b*, and Δ*E*), FAV, MDA content, SOD and CAT activities, antioxidant compounds, and DPPH radical scavenging ability at varying working pressures and treatment times. A correlation heatmap was plotted using the Pearson correlation coefficient (r^2^) (Figure 7).

As demonstrated in Figure 7, the analysis revealed significant relationships among the measured parameters. FAV (r^2^ = −0.65 and r^2^ = −0.054) and AA content (r^2^ = −0.54 and r^2^ = −0.47) exhibited negative correlations with working pressure and treatment time, respectively. In contrast, a* (r^2^ = 0.039 and r^2^ = 0.14), SOD (r^2^ = 0.17 and r^2^ = 0.27), TPC (r^2^ = 0.61 and r^2^ = 0.50), and DPPH (r^2^ = 0.11 and r^2^ = 0.24) demonstrated positive correlations with the working pressure and treatment time, respectively. Working pressure was positively correlated with CAT activity (r^2^ = 0.44) and GSH content (r^2^ = 0.61). The DPPH radical scavenging ability exhibited positive correlations with SOD activity (r^2^ = 0.63), CAT activity (r^2^ = 0.46), TPC (r^2^ = 0.088), AA content (r^2^ = 0.22), and GSH content (r^2^ = 0.57). These findings suggest that enhanced antioxidant capacity is closely associated with increased activities of antioxidant enzymes and elevated levels of antioxidant compounds. Conversely, notable negative correlations were observed between the DPPH radical scavenging ability and both FAV (r^2^ = −0.49) and MDA (r^2^ = −0.15). These results suggest that improved antioxidant capacity is associated with reduced membrane damage and quality deterioration in maize kernels.

### 3.8. Application Potential of Cold Plasma as a Pretreatment Technology for Postharvest Grain Storage

As previously reported, FAV and MDA content are reliable indicators of maize quality deterioration during storage, given that lipid hydrolysis and oxidation advance more rapidly than protein or starch degradation [75,76]. Untreated maize samples stored at 25 °C (11.8% moisture content) for 120 days demonstrated comparable FAV levels (~40.00 mg NaOH/100 g/40.00 mg KOH/100 g) to those stored at 20 °C in silo bags (15.5% moisture) for the same duration [77]. In contrast, CP-treated samples exhibited markedly reduced FAVs: 19.42 mg NaOH/100 g (Ar plasma) and 19.49 mg NaOH/100 g (N_2_ plasma). The FAV and MDA contents in CP-treated maize samples were also lower than those reported for rice stored under conventional 30 °C conditions or a 98.9% N_2_-controlled atmosphere for 60 days [78]. Furthermore, the initial FAV and MDA levels of CP-treated maize were lower than those of the rice samples mentioned above. These results suggest that CP effectively delays quality deterioration by inhibiting lipid hydrolysis and oxidation.

The glow discharge cold plasma system—comprising four essential components (vacuum system, glow discharge generator, conveying system, and standardized material cylinders)—demonstrates significant scalability potential. The industrial-scale implementation could be achieved through the (i) parallel arrangement of many discharge units, (ii) optimization of the vacuum system’s energy consumption, and (iii) integration with continuous grain conveying systems. Notably, differences in physicochemical properties and antioxidant capacity between Ar and N_2_ plasma-treated maize samples were observed, likely attributed to distinct energy levels of reactive species generated by different gas sources. Under identical discharge voltage and treatment duration, Ar plasma produced stronger current and higher-energy active species than N_2_ plasma [79], correlating with its 13.09% superior DPPH radical scavenging efficacy after 180 days of storage. However, given the higher cost of Ar and the considerable preservation of sensory qualities, N_2_ plasma treatment may be more cost-effective for practical applications.

## 4. Conclusions

The present study validates glow discharge cold plasma as an effective non-thermal strategy for maize quality preservation through dual mechanisms of antioxidant enhancement and oxidative suppression. The optimized treatment (100 Pa working pressure and 5 min exposure) significantly fortified the antioxidant defense system, evidenced by 38.46–99.10% increases in SOD/CAT activity, approximately 16.00% elevation in TPC, and enhanced AA and GSH levels, alongside 54.47–56.37% and 30.00–45.00% reductions in FAV and MDA content, respectively. These coordinated modifications sustained enhanced antioxidant capacity throughout 180 days of ambient storage, with significantly lower FAV and MDA rebound compared to untreated controls, demonstrating CP’s efficacy in prolonging storage stability. While CP demonstrates superior preservation efficacy compared to conventional methods, its commercial implementation requires systematic pilot-scale validation of nutrient retention and sensory attributes. To advance this technology toward industrial adoption, future research should prioritize (i) deciphering the molecular mechanisms underlying CP-induced effects through integrated transcriptomic–metabolomic profiling, and (ii) developing hybrid preservation systems combining CP with controlled-atmosphere storage technologies to achieve synergistic quality stabilization.

## Figures and Tables

**Table 1:** Color properties of different CP-treated maize kernels.

Gas	Samples	L*	a*	b*	Δ*E*	C*	h*
Ar	Untreated (0 min)	57.74 ± 0.75 ^b^	9.00 ± 0.21 ^a^	20.91 ± 0.54 ^e^	-	22.77 ± 0.53 ^d^	1.16 ± 0.02 ^a^
	75-1 m	59.29 ± 0.73 ^ab^	8.19 ± 0.12 ^bcd^	22.80 ± 0.30 ^cd^	2.66	24.22 ± 0.35 ^c^	1.23 ± 0.01 ^a^
	75-5 m	59.08 ± 0.76 ^ab^	7.87 ± 0.16 ^cd^	21.97 ± 0.49 ^d^	2.17	23.44 ± 0.56 ^d^	1.23 ± 0.01 ^a^
	75-10 m	59.03 ± 0.81 ^ab^	8.29 ± 0.21 ^bc^	23.34 ± 0.46 ^bc^	2.94	24.77 ± 0.45 ^bc^	1.23 ± 0.02 ^a^
	100-1 m	58.66 ± 0.74 ^ab^	8.11 ± 0.04 ^bcd^	23.90 ± 0.37 ^b^	3.33	25.24 ± 0.44 ^ab^	1.24 ± 0.00 ^a^
	100-5 m	60.05 ± 0.79 ^a^	7.77 ± 0.32 ^d^	24.92 ± 0.51 ^a^	4.87	26.10 ± 0.71 ^a^	1.27 ± 0.01 ^a^
	100-10 m	58.63 ± 0.56 ^ab^	8.49 ± 0.27 ^b^	23.92 ± 0.48 ^b^	3.23	25.39 ± 0.58 ^ab^	1.23 ± 0.01 ^a^
	125-1 m	58.76 ± 0.60 ^ab^	8.19 ± 0.13 ^bcd^	23.70 ± 0.40 ^bc^	3.13	25.07 ± 0.51 ^bc^	1.24 ± 0.00 ^a^
	125-5 m	59.83 ± 0.36 ^a^	8.05 ± 0.24 ^bcd^	23.41 ± 0.51 ^bc^	3.45	24.75 ± 0.68 ^bc^	1.24 ± 0.00 ^a^
	125-10 m	58.84 ± 0.68 ^ab^	8.36 ± 0.27 ^bc^	23.22 ± 0.05 ^bc^	2.73	24.68 ± 0.13 ^bc^	1.23 ± 0.01 ^a^
N_2_	Untreated (0 min)	57.74 ± 0.75 ^b^	9.00 ± 0.21 ^a^	20.91 ± 0.54 ^e^	-	22.77 ± 0.53 ^e^	1.16 ± 0.02 ^e^
	75-1 m	58.65 ± 0.75 ^b^	8.51 ± 0.32 ^ab^	23.98 ± 0.29 ^b^	3.35	25.45 ± 0.46 ^ab^	1.23 ± 0.01 ^bc^
	75-5 m	58.87 ± 0.62 ^b^	8.47 ± 0.20 ^ab^	22.06 ± 0.39 ^d^	1.82	23.63 ± 0.53 ^d^	1.20 ± 0.00 ^d^
	75-10 m	58.55 ± 0.79 ^b^	8.39 ± 0.19 ^b^	22.35 ± 0.07 ^d^	1.91	23.88 ± 0.16 ^d^	1.21 ± 0.01 ^cd^
	100-1 m	59.04 ± 0.66 ^b^	8.14 ± 0.22 ^b^	23.51 ± 0.34 ^bc^	3.12	24.88 ± 0.48 ^bc^	1.24 ± 0.01 ^b^
	100-5 m	61.58 ± 0.66 ^a^	7.94 ± 0.27 ^b^	24.80 ± 0.16 ^a^	5.61	26.05 ± 0.19 ^a^	1.26 ± 0.01 ^a^
	100-10 m	58.70 ± 0.61 ^b^	8.06 ± 0.26 ^b^	23.61 ± 0.26 ^bc^	3.06	24.95 ± 0.19 ^bc^	1.24 ± 0.02 ^ab^
	125-1 m	59.22 ± 0.48 ^b^	8.15 ± 0.33 ^b^	23.65 ± 0.05 ^bc^	3.27	25.02 ± 0.08 ^bc^	1.24 ± 0.02 ^ab^
	125-5 m	58.24 ± 0.77 ^b^	8.47 ± 0.28 ^ab^	23.85 ± 0.27 ^bc^	3.14	25.31 ± 0.39 ^bc^	1.23 ± 0.01 ^bc^
	125-10 m	58.79 ± 0.61 ^b^	8.32 ± 0.23 ^b^	23.21 ± 0.17 ^c^	2.68	24.66 ± 0.23 ^c^	1.23 ± 0.01 ^bcd^

L*: lightness, a*: green (−)/red (+), b*: blue (−)/yellow (+), Δ*E*: total color difference, C*: chroma; h*: hue. Different letters in the same column indicate significant differences (*p* < 0.05) among the different samples. “X-Y min” indicates that the working pressure is X Pa for Y min.

## Data Availability

The original contributions presented in the study are included in the article, and further inquiries can be directed to the corresponding authors.
